# Auspicious Symbols of Rank and Status

**DOI:** 10.3201/eid2605.AC2605

**Published:** 2020-05

**Authors:** Byron Breedlove, Isaac Chun-Hai Fung

**Affiliations:** Centers for Disease Control and Prevention, Atlanta, Georgia, USA (B. Breedlove); Georgia Southern University, Statesboro, Georgia, USA (I.C.-H. Fung)

**Keywords:** art science connection, emerging infectious diseases, art and medicine, about the cover, public health, coronavirus, viruses, severe acute respiratory syndrome coronavirus, SARS, Middle East respiratory syndrome coronavirus, MERS, severe acute respiratory syndrome coronavirus 2, SARS-CoV-2, coronavirus disease, COVID-19, respiratory infections, One Health, Qing dynasty, buzi, Mandarin squares, auspicious symbols of rank and status, rank badge with leopard, wave and sun motifs, late 18th century, leopard, pandemic, China, zoonoses

**Figure:**
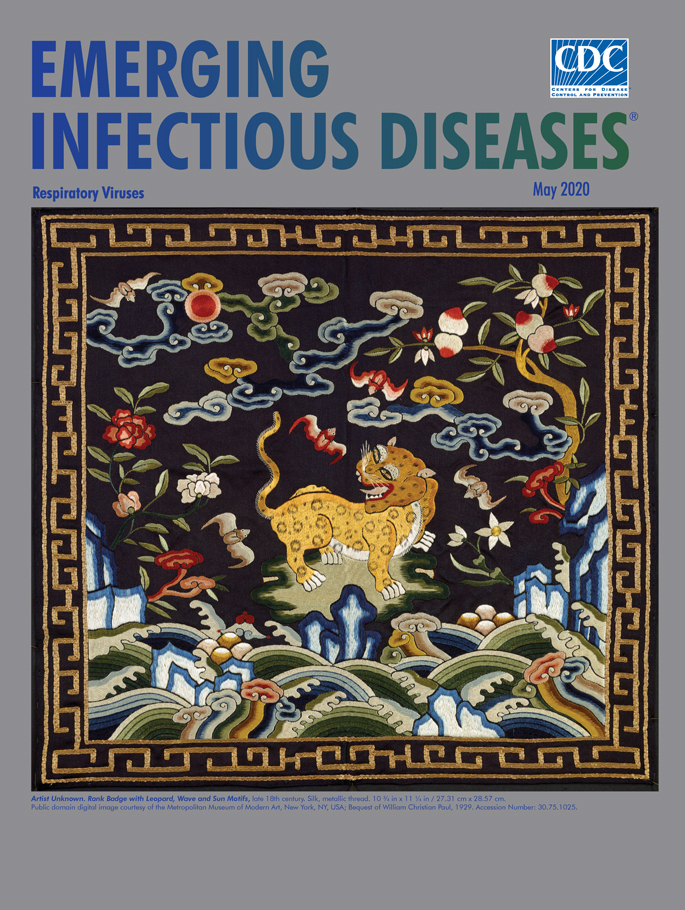
**Artist Unknown. Rank Badge with Leopard, Wave and Sun Motifs, late 18th century. Silk, metallic thread. 10 3/4 in x 11 1/4 in / 27.31 cm x 28.57 cm.** Public domain digital image courtesy of the Metropolitan Museum of Modern Art, New York, NY, USA.; Bequest of William Christian Paul, 1929. Accession no.30.75.1025.

While walking along the bustling streets of Beijing, Chengde, Shenyang, Wuhan, or other Chinese cities during the Qing dynasty (1644–1911), people would regularly brush past bats, cranes, pheasants, peacocks, egrets, or ducks; slow their step so a lion, leopard, tiger, rhinoceros, or bear could hurry past; or yield to allow passage to a dragon, unicorn, or *qilin* (a chimera with horns, a dragon’s head, fish scales, an oxen’s tail, horse’s hooves, and multicolored skin). Of course, it was not those actual animals jostling their way through the crowded causeways but rather myriad Chinese statesmen, civic officials, military officers, and members of the imperial court, as well as their wives, all of whom indicated their rank and status by wearing embroidered badges featuring images of those creatures on their outer coats. 

From the late 14th century until the early 20th century ce, these ornate rank badges (called *buzi* or Mandarin squares) featured fierce animals to denote military officials, various bird species to identify civic officials, and exotic and imaginary creatures to signify members of the imperial court. Art historian Mary Dusenbury writes, “Qing badges generally include an abbreviated cosmic diagram with an earth-mountain in the lower center, and a multitude of auspicious symbols filling up the surrounding space. In the center, the animal or bird looks up at a prominent red sun, symbol of the emperor.”

This month’s cover image is an 18th century Qing military rank badge that depicts a muscular leopard standing on a small piece of light brown, green-tinged land amidst flowering plants and fruit trees. The surrounding sky is filled with swooping bats and tendrils of clouds. Stylized ocean waves rise and swell, and breakers reach out like fingers on a hand. What may be *lingzhi* mushrooms, which are used in traditional Chinese medicine and are also symbols of immortality, sprout from the junction of ocean with land. The small red sun disk in the upper left represents the emperor and provides a focal point for the leopard. The leopard itself symbolizes power, important for military officers. Combined, the various elements related to the sky, sea, and land denote the universe, and the bats indicate good fortune. The bold colors and textures of the finely woven threads stand out in contrast to the black background, and an intricately designed gold border wraps the edges of the badge.

According to the University of Michigan Museum of Art, “During the Ming dynasty, the leopard and tiger shared the third and fourth ranks and in the early years of the Qing dynasty the tiger was the third rank. From 1662 to the end of the Qing dynasty, the leopard solely represented the third military rank.” 

Other changes in rank, which correspond to the start of Emperor Kangxi's reign, included assigning the *qilin* and lion to represent the first and second military ranks, respectively―both ranks had previously been symbolized by lions. These woven or embroidered badges could be removed and replaced should the official or office be promoted to a higher rank. Although the design of the squares evolved from one dynasty to the next―for instance, Qing badges were smaller than Ming badges and featured decorative borders―the specific birds and animals used to denote rank remained more or less constant. 

The birds and animals featured on the various rank badges (excepting, among others, dragons, unicorns, and *qilin*) may also serve as zoonotic reservoirs capable of transmitting viral pathogens that can cause respiratory infections in humans. For example, some of the birds that signify ranks among civic officials can transmit highly pathogenic avian influenza viruses. Bats, a mainstay on many badges because of their association with good fortune, are reservoirs for Hendra and Nipah viruses and for the severe acute respiratory syndrome (SARS) coronavirus. Coronaviruses are also found in many different species of animals besides bats, including swine, camels, and cattle. 

The current COVID-19 pandemic is caused by a coronavirus named SARS-CoV-2. Many factors affect interactions among humans, animals, plants, and the environment, creating greater opportunities for novel pathogens, such as SARS-CoV-2 to emerge. Perhaps one’s rank or status might confer favor in some circles, but they offer no protection from human-to-human transmission of SARS-CoV-2 and other viral respiratory infections and illnesses.
